# Myocardial Work Assessment for the Prediction of Prognosis in Advanced Heart Failure

**DOI:** 10.3389/fcvm.2021.691611

**Published:** 2021-06-18

**Authors:** Felix Hedwig, Olena Nemchyna, Julia Stein, Christoph Knosalla, Nicolas Merke, Fabian Knebel, Andreas Hagendorff, Felix Schoenrath, Volkmar Falk, Jan Knierim

**Affiliations:** ^1^Department of Cardiothoracic and Vascular Surgery, German Heart Center Berlin, Berlin, Germany; ^2^DHZB Dienstleistungs GmbH, Berlin, Germany; ^3^DZHK (German Centre for Cardiovascular Research), Partner Site Berlin, Berlin, Germany; ^4^Department of Cardiology and Angiology, Charité – Universitätsmedizin Berlin, corporate member of Freie Universität Berlin, Humboldt-Universität zu Berlin, and Berlin Institute of Health, Berlin, Germany; ^5^Department of Cardiology, Klinik und Poliklinik für Kardiologie, University of Leipzig, Leipzig, Germany; ^6^Department of Cardiovascular Surgery, Charité – Universitätsmedizin Berlin, corporate member of Freie Universität Berlin, Humboldt-Universität zu Berlin, and Berlin Institute of Health, Berlin, Germany; ^7^Eidgenössische Technische Hochschule Zürich, Department of Health Sciences and Technology, Translational Cardiovascular Technology, Zurich, Switzerland

**Keywords:** myocardial work, prognosis, strain, heart failure, outcome, constructive work

## Abstract

**Objectives:** The aim of this study was to investigate whether echocardiographic assessment of myocardial work is a predictor of outcome in advanced heart failure.

**Background:** Global work index (GWI) and global constructive work (GCW) are calculated by means of speckle tracking, blood pressure measurement, and a normalized reference curve. Their prognostic value in advanced heart failure is unknown.

**Methods:** Cardiopulmonary exercise testing and echocardiography with assessment of GWI and GCW was performed in patients with advanced heart failure caused by ischemic heart disease or dilated cardiomyopathy (*n* = 105). They were then followed up repeatedly. The combined endpoint was all-cause death, implantation of a left ventricular assist device, or heart transplantation.

**Results:** The median patient age was 54 years (interquartile range [IQR]: 48–59.9). The mean left ventricular ejection fraction was 27.8 ± 8.2%, the median NT-proBNP was 1,210 pg/ml (IQR: 435–3,696). The mean GWI was 603 ± 329 mmHg% and the mean GCW was 742 ± 363 mmHg%. The correlation between peak oxygen uptake and GWI as well as GCW was strongest in patients with ischemic cardiomyopathy (*r* = 0.56, *p* = 0.001 and *r* = 0.53, *p* = 0.001, respectively). The median follow-up was 16 months (IQR: 12–18.5). Thirty one patients met the combined endpoint: Four patients died, eight underwent transplantation, and 19 underwent implantation of a left ventricular assist device. In the multivariate Cox regression analysis, only NYHA class, NT-proBNP and GWI (hazard ratio [HR] for every 50 mmHg%: 0.85; 95% CI: 0.77–0.94; *p* = 0.002) as well as GCW (HR for every 50 mmHg%: 0.86; 95% CI: 0.79–0.94; *p* = 0.001) were identified as independent predictors of the endpoint. The cut-off value for predicting the outcome was 455 mmHg% for GWI (AUC: 0.80; *p* < 0.0001; sensitivity 77.4%; specificity 71.6%) and 530 mmHg% for GCW (AUC: 0.80; *p* < 0.0001; sensitivity 74.2%; specificity 78.4%).

**Conclusions:** GWI and GCW are powerful predictors of outcome in patients with advanced heart failure.

## Introduction

Identifying patients with advanced heart failure and a high-risk prognosis at an early stage is paramount for the appropriate timing and type of treatment (1, 2). Risk models, cardiopulmonary exercise tests (CPX), and some biomarkers have been shown to be helpful in predicting the outcome (3–5). However, applied to the population of patients with heart failure with reduced ejection fraction (HFrEF) as a whole, these tools can be time-consuming and cost-intensive.

Conventional echocardiographic parameters like left ventricular end-diastolic diameter (LVEDD), left ventricular volumes, and left ventricular ejection fraction (LVEF) are known predictors of outcome and cardiac events (6–8).

Heart failure guidelines recommend that all patients with heart failure should be routinely evaluated with transthoracic echocardiography (2) to assess systolic and diastolic ventricular function and to identify additional cardiac pathologies, such as pericardial effusion or valvular dysfunction. Furthermore, transthoracic echocardiography allows for a non-invasive assessment of left ventricular myocardial pressure strain loops. To this end myocardial deformation imaging is performed using two-dimensional speckle tracking and the afterload of the left ventricle is calculated from non-invasive brachial cuff blood pressure measurements against an empiric, normalized reference curve. These measurements have been validated against invasive measurements and were shown to accurately quantify myocardial work (9, 10).

Normal values of myocardial work parameters measured by echocardiography have been identified (11, 12). Myocardial work is reduced in patients with hypertrophic cardiomyopathy, amyloidosis, and in patients with acute coronary syndromes (13–17). It is likewise impaired in patients with HFrEF (18–20). Myocardial work indices increase under heart failure medication and can be used to improve the prediction of the response to cardiac resynchronization therapy (CRT) and the outcome in patients with HFrEF (18, 20–23).

The aim of our study was to evaluate the value of myocardial work indices in predicting the prognosis in end-stage heart failure patients with HFrEF undergoing evaluation for heart transplantation or left ventricular assist device (LVAD) implantation.

## Methods

### Patient Population and Follow-Up

Data and echocardiography images of patients who presented in the outpatient department between July 2018 and October 2019 for an evaluation of their indication for heart transplantation or LVAD implantation were reviewed retrospectively. Inclusion criteria for the study were:

- Heart failure with reduced ejection fraction (HFrEF)
- Heart failure caused by ischemic heart disease (ICM) or idiopathic dilated cardiomyopathy
- Sinus rhythm and absence of significant extrasystoles (e.g., bigeminal rhythm)
- Availability of optimal image quality for a work analysis
- Availability of blood pressure measurement immediately after echocardiography (see below)

Echocardiographic results, post-processing analysis, laboratory tests, ECG data and results of cardiopulmonary exercise testing were collected at the time of inclusion. After the baseline assessment, patients were regularly followed up in the outpatient department for monitoring of heart failure progression and treatment. Patients were listed for transplantation or underwent LVAD implantation in accordance with the current guidelines and recommendations (2, 4, 24). Myocardial work analysis was performed retrospectively (see below) it was not part of the decision making process for transplant listings or LVAD implantation. A few patients were followed up by other centers. Data of these patients were transferred with their permission. All-cause death, implantation of a left ventricular assist device, or heart transplantation were defined as the combined clinical endpoint. The study was reviewed and approved by the local ethics committee (EA2/051/19), which waived the need for written informed consent for publication of the study data.

### Echocardiography

Echocardiography was performed by experienced operators using the Vivid E9 and Vivid S70 ultrasound systems (GE Healthcare). Routine echocardiography included 2D, M-mode, and Doppler measurements as stipulated in the current guidelines (25). Particular care was taken to achieve optimal image quality. Endocardial borders and myocardium of all segments had to be clearly visualized throughout the whole cardiac circle. The images were acquired at the highest possible frame rate.

For the myocardial work analysis, patients' blood pressure was measured in a supine position immediately after the echocardiogram. As a rule, three measurements were performed and the mean systolic and diastolic pressures were used.

### Post-processing Analysis

Two-dimensional speckle-tracking analysis was performed retrospectively, offline using the EchoPac Software, Version 202. Markers for aortic valve opening and aortic valve closure were set using the PW Doppler signal of the left ventricular outflow tract. Mitral valve opening and closing time were preferably used from the PW Doppler mitral valve inflow signal. If the signal was not sufficient, the timing was set manually using the 2D image of the apical long-axis view. To measure the global longitudinal strain, the region of interest (ROI) was marked from the endocardium to the epicardium in LV-focused apical long-axis, 4-chamber, 2-chamber and 3-chamber views. Mitral annulus, left ventricular outflow tract and papillary muscles were excluded from ROI. Pressure strain loops, myocardial work, and work indices were calculated using custom software (GE Healthcare). The method has been described in detail elsewhere (9, 26). It involves a combination of left ventricular strain data recorded throughout the cardiac circle with estimated left ventricular pressure using non-invasive arterial pressure measurement and an empirical, normalized reference curve. As a result, the following indices were calculated:

- Global Work Index (GWI): Average myocardial work using strain-pressure loops from mitral valve closure to mitral valve opening
- Global Wasted Work (GWW): Work during lengthening in systole plus work during shortening in isovolumetric ventricular contraction
- Global Constructive Work (GCW): Myocardial work during shortening in systole plus myocardial work during lengthening in isovolumetric ventricular contraction.
- Global Positive Work (GPW): Myocardial work during shortening in systole plus isovolumetric ventricular contraction
- Global Systolic Constructive Work (GSCW): Myocardial work during shortening in systole

The analysis was performed by three experienced operators. The inter-observer variability of this method is known to be very good (17, 27).

### Cardiopulmonary Exercise Testing (CPX)

Where indicated, the patients performed a cardiopulmonary exercise test on the same day as the echocardiogram. CPX was performed on an upright electrical braked bicycle ergometer (AMEDTEC ECGpro; Medizintechnik Aue GmbH, Aue, Germany). A ramp protocol starting with 20 Watts and stepwise increments of 16 Watts/min was used. The pedal rate was kept steady at >45 rpm. All patients were instructed to perform at maximum effort.

CPX included continuous electrocardiographic monitoring and periodic blood pressure measurements. Gas exchange was analyzed at rest, during exercise, and during recovery with breath-by-breath measurements of oxygen uptake, carbon dioxide output, and ventilation. The test was terminated if patients exhibited signs of exhaustion, angina pectoris, significant ST-segment depression or if the maximum physical capacity was reached. The peak oxygen uptake (peak VO_2_) and ventilation-carbon dioxide output relation (VE/VCO_2_) slope were measured according to the current guidelines (28).

### Statistical Analysis

Continuous data are presented as mean and standard deviation or as median and interquartile range, as appropriate. Categorical data are summarized as absolute and relative frequencies. Patient groups were compared using the *t*-test or the Wilcoxon-Mann-Whitney test for continuous variables and Fisher's exact test or the chi-square test for categorical variables. Correlations were calculated using Pearson's correlation coefficient. A receiver operating characteristic (ROC) analysis was performed to identify cut-offs for predicting the outcome. Univariate and multivariate Cox proportional hazard regression analyses were applied to assess predictors of adverse outcomes. For multivariate analysis we focused on clinical and echocardiographic parameters of known prognostic relevance in heart failure. For the variable selection, the Least Absolute Shrinkage and Selection Operation (LASSO) (29) was used to overcome the small number of observations and events. This selection process was performed twice, each time considering either GWI or GCW. A Kaplan-Meier analysis was carried out to estimate the differences in outcome between the groups.

Analyses were exploratory in nature. For statistical calculations, we used R version 4.0.2 software (R Foundation for Statistical Computing, Vienna, Austria) and SPSS, version 25 (SPSS, Chicago, IL, USA).

## Results

### Patient Population

One hundred and sixty echocardiograms of patients who presented between July 2018 and October 2019 for evaluation of the indication for heart transplantation or LVAD implantation were reviewed. Fifty patients had to be excluded because of irregular heart rhythm (*n* = 11), poor image quality (*n* = 31), or other cause of heart failure (*n* = 8). Furthermore, five patients were excluded because of missing results of the blood pressure measurement. Thus, 105 patients were included in the study. Their median age was 54 years (IQR: 48–59.5 years); 80% (*n* = 84) were male. 40% (*n* = 42) had ischemic heart disease and 60% (*n* = 63) had idiopathic dilated cardiomyopathy. All patients received optimal medical heart failure therapy as per the current guidelines (2). For baseline characteristics see Table 1.

**Table 1:** Clinical and demographic characteristics at baseline.

	**All patients (*n* = 105)**	**Patients who met the endpoint (*n* = 31)**	**Patients who did not meet the endpoint (*n* = 74)**	***p*-value**
Age (years) (median [IQR])	54 [48–59.5]	58 [48–63]	54 [48–58]	0.123
Gender				0.79
Female	21 (20)	7 (22.6)	14 (18.9)	–
Male	84 (80)	24 (77.4)	60 (81.1)	–
Body mass index (kg/m^2^)	28.4 ± 4.6	28.3 ± 4.1	28.4 ± 4.8	0.96
**Blood pressure (mmHg)**				
Systolic	106.5 ± 18.1	97.7 ± 14.3	110.2 ± 18.3	0.001
Diastolic	66.1 ± 12.5	60.5 ± 13.2	68.5 ± 11.5	0.002
NYHA class				<0.0001
NYHA II	45 (42.9)	3 (9.7)	42 (56.8)	–
NYHA III	55 (52.4)	25 (80.6)	30 (40.5)	–
NYHA IV	5 (4.8)	3 (9.7)	2 (2.7)	–
Etiology of HF				0.83
DCM	63 (60)	18 (58.1)	45 (60.8)	–
ICM	42 (40)	13 (41.9)	29 (39.2)	–
Peripheral Edema	16 (15.2)	10 (32.3)	6 (8.1)	0.005
HF known (months) (median [IQR])	48 [15–122.5]	96 [36–144]	31.5 [11.3–96]	0.1
NT-proBNP, pg/dl (median [IQR])	1,210 [435–3,696]	5,900 [4,053–9,076]	1,297 [589–3,544]	<0.0001
**Devices**				
ICD	43 (41)	16 (51.6)	27 (36.5)	0.19
CRT ± D	40 (38.1)	13 (41.9)	27 (36.5)	0.66
**Bundle branch block**				
LBBB	14 (13.3)	6 (19.4)	8 (10.8)	0.34
BBB caused by pacemaker	36 (34.3)	13 (41.9)	23 (31.1)	0.37
**Medication**				
Beta-blocker	98 (93.3)	27 (87.1)	71 (95.9)	0.19
ACE-I	20 (19)	4 (12.9)	16 (21.6)	0.42
ARB	13 (12.4)	4 (12.9)	8 (12.2)	1.0
ARNI	71 (67.6)	21 (67.7)	50 (67.6)	1.0
Aldosterone antagonist	90 (85.7)	25 (80.6)	65 (87.8)	0.37
Loop diuretic	90 (85.7)	30 (96.8)	60 (81.1)	0.037
**Cardiopulmonary test**				
VO_2_ peak, ml/min/kg	11.9 ± 5.0	9.1 ± 2.6	13.2 ± 5.3	<0.0001
VE/VCO_2_ slope, l/l (median [IQR])	34 [29–41]	41 [36–46.5]	31 [28–37]	<0.0001

*Values are given as a number (percent) or mean ± standard deviation except where otherwise indicated*.

*IQR, interquartile range; NYHA, New York Heart Association; DCM, dilated cardiomyopathy; ICM, heart failure caused by ischemic heart disease; BNP, brain natriuretic peptide, ICD, implantable cardioverter-defibrillator; CRT ± D, cardiac resynchronization therapy with or without defibrillator; LBBB, left bundle branch block; ACE-I, angiotensin-converting enzyme inhibitor; ARB, angiotensin II receptor blocker; ARNI, angiotensin-receptor neprilysin inhibitor; peak VO_2_, peak oxygen uptake; VE/VCO_2_, ventilation-carbon dioxide output relation*.

### Echocardiography

At baseline, all patients had severe left ventricular dilatation with a mean end-diastolic volume index of 109 ± 39 ml/m2. The mean left ventricular ejection fraction was 27.8 ± 8.2%. Diastolic function was impaired, with an average E/e' of 17.5 ± 8.8. The systolic pulmonary artery pressure, calculated from the peak tricuspid regurgitation velocity, was 30.4 ± 11.8 mmHg. In 13.3% (*n* = 14) of the patients, the severity of mitral regurgitation was more than moderate. The complete results of the standard transthoracic echocardiographic exams are shown in Table 2.

**Table 2:** Echocardiographic characteristics of all patients.

	**All patients (*n* = 105)**	**Patients who met the endpoint (*n* = 31)**	**Patients who did not meet the endpoint (*n* = 74)**	***p*-value**
LVEDD (mm)	63.8 ± 9.4	68.5 ± 7.8	61.9 ± 9.4	0.001
LVEDDI (mm/m^2^)	31.1 ± 5.1	33.2 ± 5	30.3 ± 5	0.006
FS (%)	12.8 ± 8	10.3 ± 9.1	13.3 ± 7.4	0.076
LVEDV—Simpson (ml)	227 ± 88	246 ± 64	218 ± 95	0.14
LVEDVI—Simpson (ml/m^2^)	109 ± 39	119 ± 32	105 ± 41	0.09
LVEF—Simpson (%)	27.8 ± 8.2	22.2 ± 5.8	30.1 ± 8	<0.0001
LVOT VTI (cm)	14.8 ± 4.1	11.8 ± 2.5	16 ± 3.9	<0.0001
SV—LVOT (ml)	53.1 ± 14.8	42.5 ± 11.6	57.5 ± 13.7	<0.0001
Mitral regurgitation				0.001
None	25 (23.8)	2 (6.5)	23 (31.1)	
Mild	45 (42.9)	11 (35.5)	34 (45.9)	
Moderate	21 (20)	9 (29)	12 (16.2)	
Severe	14 (13.3)	9 (29)	5 (6.8)	
Tricuspid regurgitation				0.14
None	49 (46.7)	11 (35.5)	38 (51.4)	
Mild	39 (37.1)	12 (38.7)	27 (36.5)	
Moderate	11 (10.5)	4 (12.9)	7 (9.5)	
Severe	6 (6)	4 (12.9)	2 (2.7)	
PA pressure (mmHg)	30.4 ± 11.8	34 ± 11.5	28.5 ± 11.6	0.1
TAPSE (mm)	19.9 ± 4.5	18 ± 3.5	20.7 ± 4.6	0.005
E-velocity (m/s)	0.83 ± 0.3	0.97 ± 0.3	0.77 ± 0.3	0.002
E/e' average	17.5 ± 8.8	21.6 ± 9	15.7 ± 8.1	0.002
Deceleration time (ms)	159 ± 64	142 ± 66	167 ± 62	0.081
Global longitudinal strain (%)	−7.1 ± 3.2	−4.9 ± 2.1	−7.97 ± 3.2	<0.0001
GWE (mmHg%)	76.2 ± 10.3	72.2 ± 8.3	77.9 ± 10.6	0.009
GWI (mmHg%)	603 ± 329	378 ± 173	697 ± 334	<0.0001
GCW (mmHg%)	742 ± 363	497 ± 210	845 ± 366	<0.0001
GWW (mmHg%)	164 ± 92	143 ± 69	173 ± 99	0.13
GPW (mmHg%)	755 ± 357	508 ± 210	858 ± 355	<0.0001
GSCW (mmHg%)	695 ± 339	467 ± 198	791 ± 341	<0.0001

*Values are given as a number (percent) or mean ± standard deviation except where otherwise indicated*.

*LVEDD, left ventricular end-diastolic diameter; LVEDDI, left ventricular end-diastolic diameter index; LVEDV, left ventricular end-diastolic volume; LVEDVI, left ventricular end-diastolic volume index; LVEF, left ventricular ejection fraction; LVOT VTI, left ventricular outflow tract velocity time integral; SV—LVOT, stroke volume calculated by continuity equation; PA, pulmonary artery calculated from peak tricuspid regurgitation velocity; TAPSE, tricuspid annular plane systolic excursion; GWE, global work efficiency; GWI, global work index; GCW, global constructive work; GWW, global wasted work; GPW, global positive work; GSCW, global systolic constructive work*.

### Post-processing Strain and Work Analysis

All patients showed reduced strain and work parameters. The mean global longitudinal strain was −7.1 ± 3.2%. The mean global work index (GWI) was 603 ± 329 mmHg%, and mean global constructive work (GCW) was 742 ± 363 mmHg%. Work efficiency was impaired (76.2 ± 10.3%). See Table 2 for details. There was no significant difference in myocardial work parameters between patients with a CRT device and without a CRT device. See Supplementary Table 2.

### Cardiopulmonary Exercise Testing (CPX)

**Figure 1:**
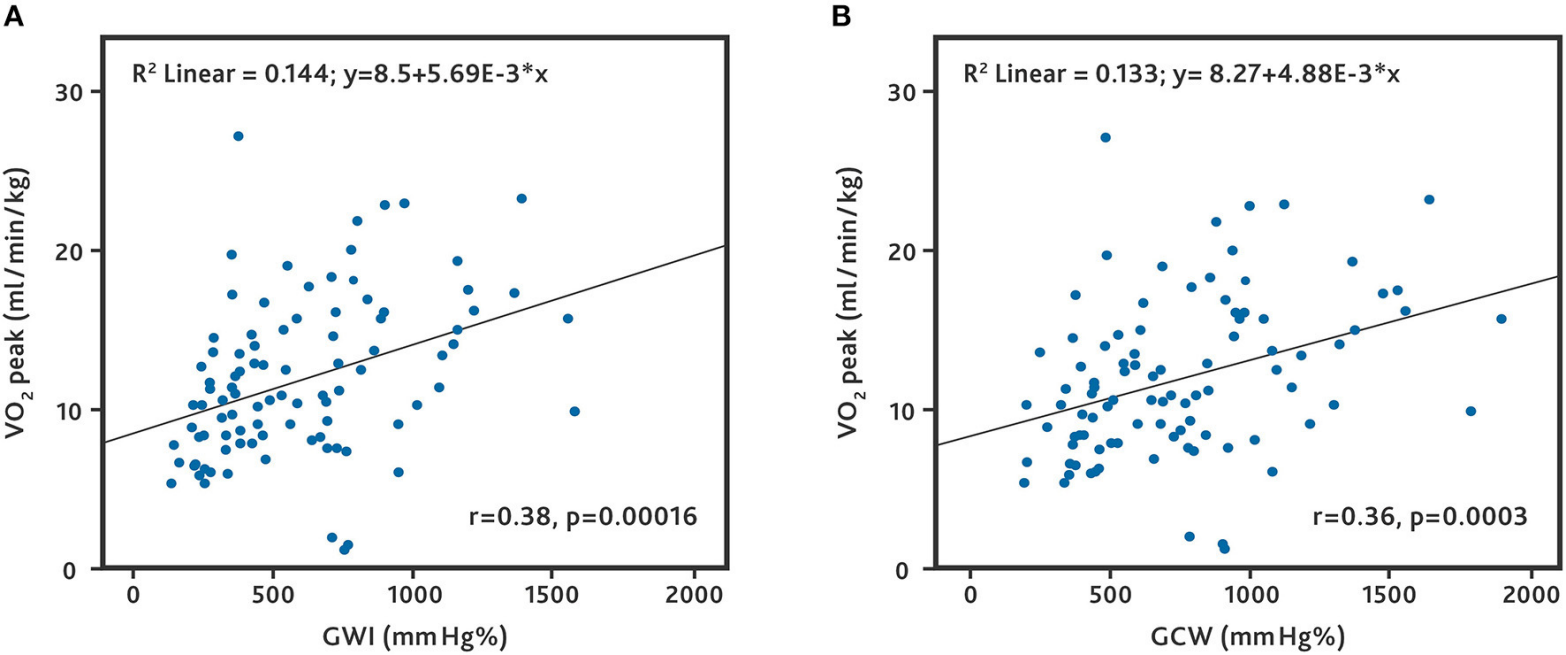
Correlation between peak oxygen uptake and global work parameters. All patients who underwent cardiopulmonary exercise testing. **(A)** Correlation between peak oxygen uptake (VO_2_ peak) and global work index (GWI). **(B)** Correlation between VO_2_ peak and global constructive work (GCW).

89.5% (*n* = 94) patients underwent cardiopulmonary exercise testing. The mean VO_2_ peak was 11.9 ± 5.0 ml/min/kg. The median VE/VCO_2_ slope was 34 l/l (IQR: 29–41 l/l). We found a correlation between VO_2_ and the parameters of the global work analysis. The correlation between VO_2_ and GWI (*r* = 0.38, *p* = 0.00016) and between VO_2_ and GCW (*r* = 0.36, *p* = 0.0003) was weak (Figure 1). When separated by the etiology of HF, patients with ICM demonstrated a higher correlation of VO_2_ with GWI and GCW (*r* = 0.56, *p* = 0.001 and *r* = 0.53, *p* = 0.001, respectively) (Supplementary Figure 1). Patients with DCM were significantly younger and exhibited higher myocardial work parameters compared to those with ICM. Patients with DCM and ICM did not differ in gender, NYHA class, most classic echocardiographic parameters, and medication (Supplementary Table 1).

### Follow-Up and Outcome

The median follow-up of all patients was 16 months (IQR: 12–18.5 months). Thirty one patients (29.5%) met the combined endpoint during follow-up: 18 patients with DCM (28.6%) and 13 patients with ICM (31%). Of these, four patients died (3.8%, only ICM patients), eight underwent transplantation (7.6%, only DCM patients), and 19 received an LVAD (18.1%, 10 DCM and 9 ICM patients). See Supplementary Table 1.

The overall 1-year event-free survival (death, LVAD implantation, heart transplantation) was 76.9% [95% confidence interval (CI): 67.6 to 83.9%]; the event-free survival at 18 months was 68.9% (95% CI: 58.6–77.1%).

For a comparison of baseline characteristics and echocardiographic measures between patients who did or did not meet the combined endpoint, see Tables 1, 2.

According to the univariate regression analysis, NYHA class, plasma levels of NT-proBNP, LVEF, left ventricular diastolic function (E/e' average), TAPSE, GLS, GWI, and GCW were predictors of the combined outcome (Table 3).

**Table 3:** Univariate and multivariate Cox regression analysis for the prediction of combined outcome.

**Variables in the equation**	**Univariate**		**Multivariate for GWI**		**Multivariate for GCW**	
	**HR (95% CI)**	***p*-value**	**HR (95% CI)**	***p*-value**	**HR (95% CI)**	***p*-value**
Age, years	1.01 (0.98–1.05)	0.549				
NYHA class ≥3	9.3 (2.8–30.7)	0.0002	3.68 (1.03–13.07)	0.044	4.19 (1.22–14.37)	0.023
NT-proBNP, 500 pg/dl	1.03 (1.01–1.04)	<0.0001	1.02 (1.00–1.03)	0.012	1.02 (1.00–1.03)	0.019
LVEF, %	0.9 (0.86–0.94)	<0.0001				
LVEDVI, mL/m^2^	1.01 (0.99–1.01)	0.084				
E/E', average	1.06 (1.02–1.09)	0.001				
TAPSE, mm	0.89 (0.81–0.97)	0.006				
GLS, %	1.44 (1.23–1.68)	<0.0001				
GCW, 50 mmHg%	0.82 (0.76–0.89)	<0.0001			0.86 (0.79–0.90)	0.001
GWI, 50 mmHg%	0.81 (0.74–0.9)	<0.0001	0.85 (0.77–0.94)	0.002		

*NYHA, New York Heart Association; NT-proBNP, N-terminal pro b-type natriuretic peptide; LVEF, left ventricular ejection fraction; LVEDVI, left ventricular end-diastolic volume index; TAPSE, tricuspid annular plane systolic excursion; GLS, global longitudinal strain; GCW, global constructive work; GWI, global work index*.

According to the multivariate Cox regression analysis for the prediction of the combined endpoint, including the 10 parameters listed in Table 3, only NYHA class, NT-proBNP, and GWI or GCW had a significant influence on the outcome.

In this model each increase in GWI by 50 mmHg% resulted in an HR of 0.85 (95% CI: 0.77–0.94, *p* = 0.002), and each increase in GCW by 50 mmHg% resulted in an HR of 0.86 (95% CI: 0.79–0.94, *p* = 0.001); see Table 3.

**Figure 2:**
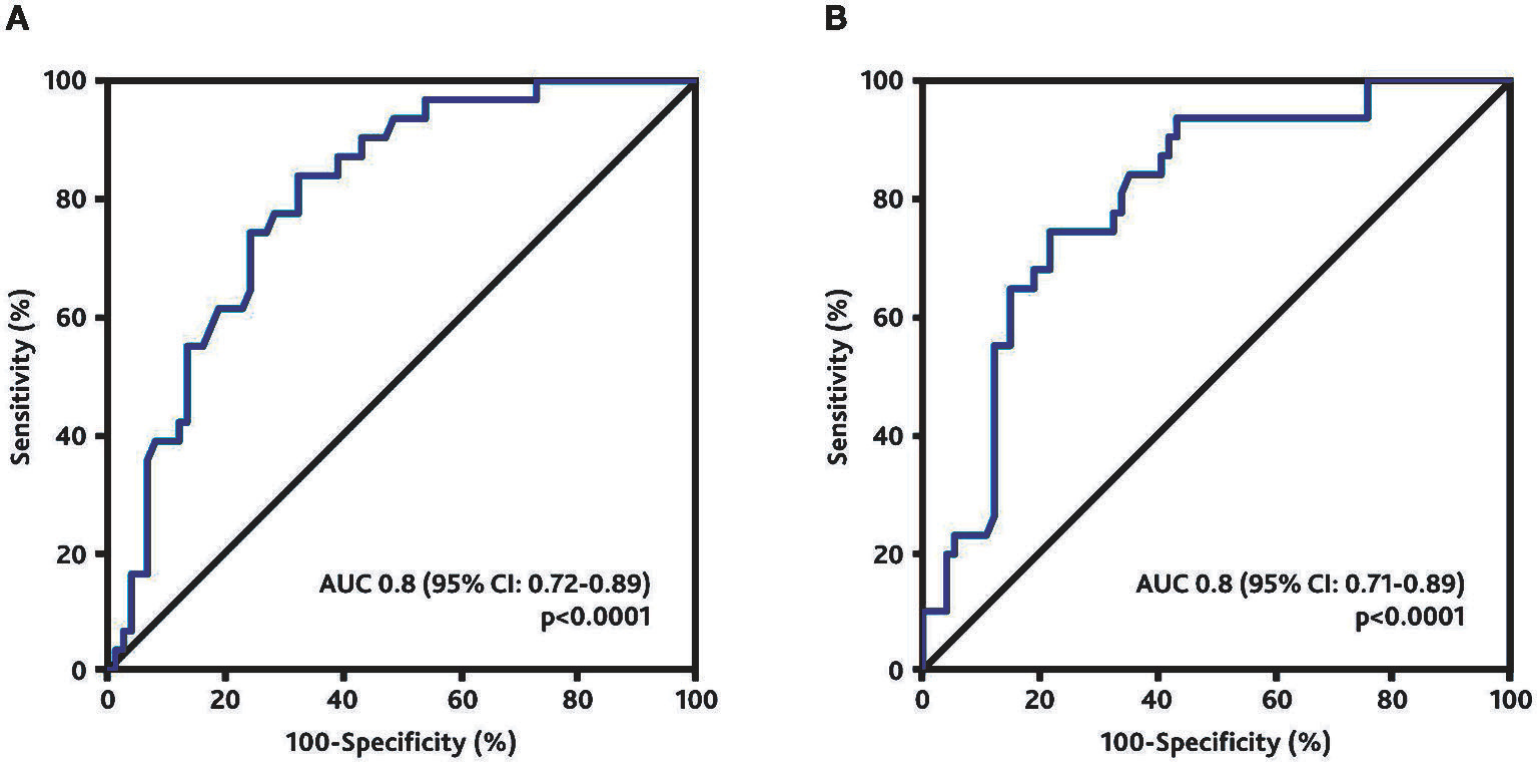
Receiver operating characteristic curves of global work parameters for the combined endpoint. **(A)** Receiver operating characteristic curve for global work index. **(B)** Receiver operating characteristic curve for global constructive work.

The GWI cut-off of 455 mmHg% was shown to predict the combined endpoint with a sensitivity of 77.4% and a specificity of 71.6% (AUC 0.8, *p* < 0.0001). The GCW cut-off of 530 mmHg% was found to predict the combined endpoint with a sensitivity of 74.2% and a specificity of 78.4% (AUC 0.80, *p* < 0.0001). See Figure 2.

**Figure 3:**
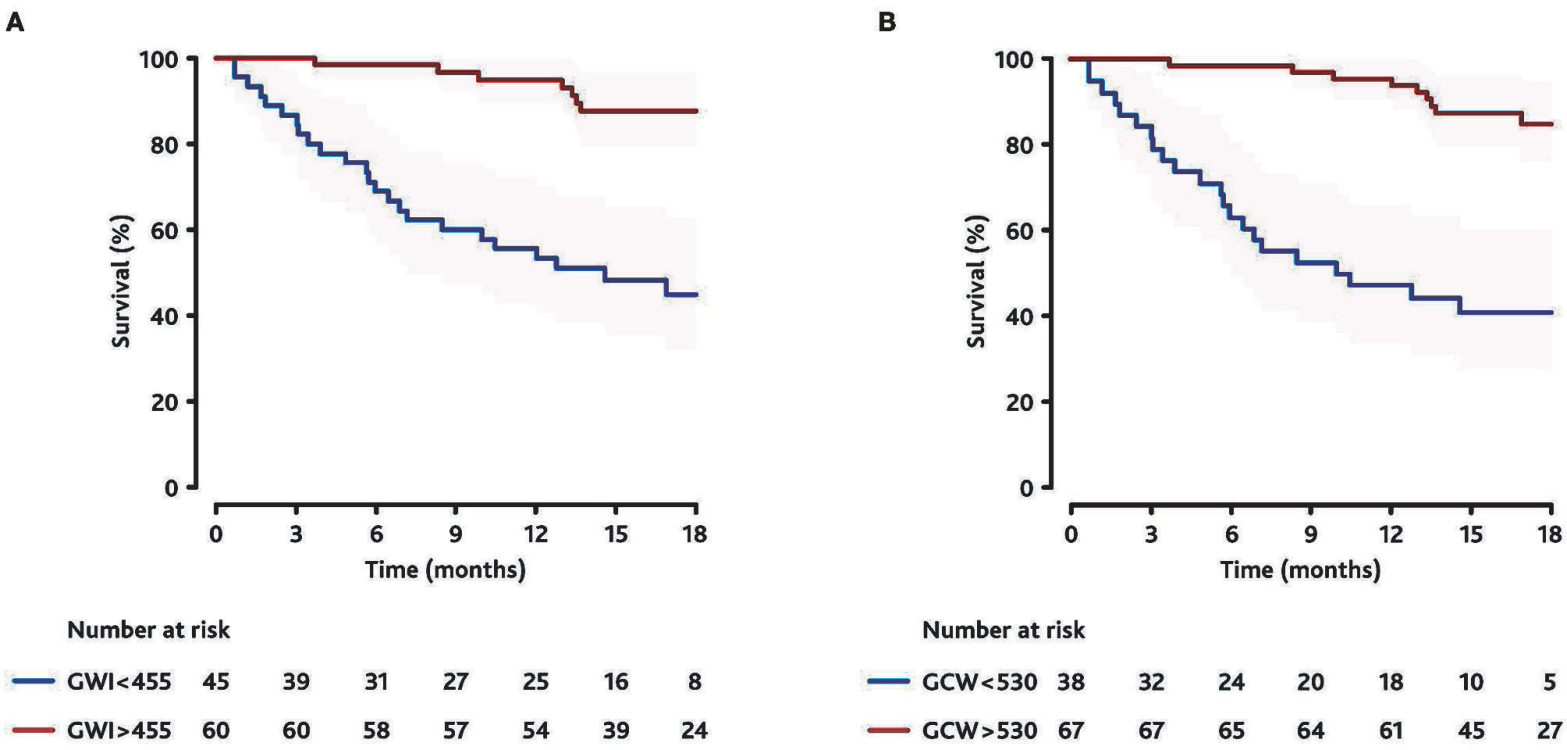
Kaplan-Meier estimates of event-free survival dependent on global work parameters. **(A)** Kaplan-Meier estimates dependent on global work index (GWI). **(B)** Kaplan-Meyer estimates dependent on global constructive work (GCW). Cut-offs derived from receiver operating curves (see Figure 2).

The Kaplan-Meier analysis showed that patients with a GWI ≥455 and patients with a GCW ≥530 had a significantly better prognosis than the control patients (see Figure 3). In patients with a GWI <455, the 1-year event-free survival rate was 53.3% (95% CI: 37.9–66.7%) and the 18-month event-free survival rate was 44.8% (95% CI: 29.3–59.1%). In patients with GWI ≥455, the 1-year event-free survival was 94.9% (95% CI: 85.0–98.3%) and the 18-month survival rate was 87.6% (95% CI: 75.6–93.9%).

In patients with a GCW <530, the 1-year event-free survival rate was 47.4% (95% CI: 31–62.1%) and the 18-month event-free survival rate was 41% (95% CI: 25.1–56.3%). In patients with a GCW ≥530, the 1-year event-free survival rate was 93.9% (95% CI: 84.5–97.7%) and the 18-month survival rate was 84.9% (95% CI: 72.6–91.9%).

## Discussion

When it comes to heart failure, identifying parameters that are associated with a rapid disease progression is paramount, as they can be used to guide therapy and follow-up management. This includes the timing for transplant listing (elective vs. high urgency) and/or implantation of a left ventricular assist device.

Transthoracic echocardiography is the method of choice for evaluating heart failure patients (2). It can be performed at the bedside without any delay or additional costs. While classic echocardiographic parameters like LVEF and left ventricular volumes are widely used for predicting the outcome, they have significant shortcomings, including inconsistency, impaired reproducibility, and a high inter-observer variability (30, 31).

Global longitudinal strain (GLS) as a quantitative method for assessing myocardial function has been shown to be superior to LVEF in predicting the outcome and has a better inter-observer variability (32–34).

Echocardiographic assessment of myocardial work may further improve the evaluation of myocardial function. This non-invasive method combines a two-dimensional strain analysis and a standardized LV pressure curve adjusted to brachial cuff pressure (9, 10). The degree of myocardial deformation is afterload-dependent, especially in patients with a severely impaired left ventricular myocardial function. Therefore, assessing global myocardial work is a very promising tool for evaluating the failing heart and predicting the prognosis.

Several global indices can be calculated with the pressure strain analysis. GWI and GCW have mostly been used to evaluate myocardial function. GWI assesses the average myocardial work from mitral valve closure to mitral valve opening, while GCW measures the work performed during shortening in systole, adding negative work during lengthening in isovolumetric relaxation. A comparison in heart failure patients showing a benefit of one parameter over the other is lacking. We evaluated both GWI and GCW and did not find a relevant difference in predictive power (see Figures 1–3). Larger studies may be required to identify a benefit of one parameter over the other. Until then, it appears advisable to assess both parameters to make the results of different studies comparable.

In a previous study we found a correlation between left ventricular work parameters and peak oxygen uptake (VO_2_ peak) in patients with advanced heart failure (27). This correlation has also been described in patients with cardiac amyloidosis (35). In the current study we were able to reproduce this finding in a larger population (Figure 1), albeit with a weak correlation. Interestingly, the correlation was stronger in patients with heart failure caused by ischemic heart disease (Supplementary Figure 1). In patients with idiopathic dilated cardiomyopathy and ICM, most clinical and echocardiographic parameters were comparable (Supplementary Table 1). Patients with ICM were older, which partly explains the poor survival rate, but also had lower global strain and work parameters, which may serve as new indicators for a poor prognosis.

There is growing evidence that myocardial work assessment offers incremental prognostic information in patients with HFrEF (18, 21, 23). It has also been shown that heart failure medication and CRT device implantation have an impact not only on prognosis but also on GCW (18, 20). The patients in our study were transferred by external centers for an evaluation of their indication for heart transplantation or LVAD implantation. At the time of inclusion nearly 80% had a cardiac implantable electronic device (CRT or ICD). At baseline there was no significant difference in myocardial work parameters between the patients with a CRT device and without a CRT device (Supplementary Table 2). All patients were receiving state-of-the-art heart failure medication, including beta-blockers (93%), aldosterone antagonists (86%), ACE inhibitors/angiotensin II receptor blockers (31%), or sacubitril/valsartan (68%) (Table 1). The mean GCW in our study was 742 ± 363 mmHg%, compared to 1,025 ± 442 mmHg% in the work by Galli et al. and 1,023 ± 449 mmHg% in the work by Bouali et al. (18) and Galli et al. (20). Our GWI was 603 ± 329 mmHg% compared to 731 ± 392 mmHg% in the study by Wang et al. (23). Together with a known duration of heart failure of 48 (IQR: 15-122.5) months, this is indicative of a population with end-stage chronic heart failure. A timely decision regarding the further surgical treatment is crucial, especially in these patients.

In the multivariate Cox regression analysis we focused on echocardiographic parameters including ejection fraction, global longitudinal strain (E/é), and end-diastolic volume in order to assess the additional value of echocardiographic work indices to predict the outcome in this specific population. We found that both GCW and GWI were independent predictors of the combined endpoint (Table 3). GLS and LVEF were significant predictors of outcome in the univariate but not in the multivariate analysis. This shows that LV work parameters may be more robust indicators of high risk in an end-stage heart failure population than GLS and LVEF.

A cut-off of 455 mmHg% for GWI and of 530 mmHg% for GCW was found to predict the combined endpoint with an acceptable sensitivity and specificity (Figure 2). Patients with a GWI ≤ 455 mmHg% or a GCW ≤ 530 mmHg% had a poor prognosis (Figure 3). These results underline the usefulness of echocardiographic work parameters. They may add additional information to the established assessment of patients with advanced heart failure. Further studies and the development and automated assessment of GCW and GWI during routine echocardiography may help to identify high-risk patients in future. This may prompt a referral to a center specialized in advanced heart failure therapy, with the option of heart transplantation and mechanical circulatory support.

### Limitations

Our study is a single-center, retrospective analysis with a limited sample size. Only 20% of the patients included were female. This is not unusual for patients with terminal heart failure undergoing heart transplantation or LVAD implantation (36, 37). However, it limits the reliability of the results in relation to female patients.

Many patients had to be excluded because of insufficient image quality, arrhythmias and/or missing result of blood pressure measurement during echocardiography. This is important since it may indicate a limitation of the use of myocardial work assessment in clinical routine. Furthermore, severe mitral regurgitation was present in >13% of the patients and it may have influenced the results of work assessment.

The study was conducted in a relatively homogeneous population of patients with advanced heart failure with a high number of events. This allowed us to perform a clear and significant outcome analysis. However, the multivariate analysis was limited to nine selected parameters. A prospective study including a greater number of patients with advanced heart failure is therefore highly desirable.

## Conclusion

Echocardiographic myocardial work analysis is a post-processing tool to assess myocardial performance. Our study demonstrates its usefulness as a powerful independent predictor of outcome in patients with advanced heart failure.

## Data Availability Statement

The original contributions presented in the study are included in the article/Supplementary Material, further inquiries can be directed to the corresponding author/s.

## Ethics Statement

The studies involving human participants were reviewed and approved by Charite Ethikkommission EA2/051/19. Written informed consent for participation was not required for this study in accordance with the national legislation and the institutional requirements.

## Author Contributions

FH, ON, and JK: concept/design, data collection, data analysis/interpretation, statistics, drafting of article, and approval. JS: statistics, data analysis, critical revision of article, and approval. CK, NM, FK, AH, FS, and VF: concept/design, critical revision of article, and approval. All authors contributed to the article and approved the submitted version.

## Conflict of Interest

AH receives lecture fees from GE Healthcare, Astra Zeneca, Novartis, and Pfizer. VF declares relevant financial activities outside the submitted work from Biotronik SE & Co., Abbott GmbH & Co. KG, Boston Scientific, Edwards Lifesciences, Medtronic, Berlin Heart, Novartis Pharma GmbH, JOTEC GmbH, and Zurich Heart. JS was employed by company DHZB Dienstleistungs GmbH. The remaining authors declare that the research was conducted in the absence of any commercial or financial relationships that could be construed as a potential conflict of interest.

## Supplementary Material

Supplementary Figure 1 Correlation between peak oxygen uptake and global work parameters. **(A)** Correlation between peak oxygen uptake (VO_2_ peak) and global work index (GWI). **(B)** Correlation between VO_2_ peak and global constructive work (GCW). Patients with heart failure caused by ischemic heart disease.

Supplementary Table 1 Clinical and echocardiographic parameters of patients with cardiac resynchronization therapy (CRT) device and without CRT device.

Supplementary Table 2 Demographic, clinical, echocardiographic, and outcome data according to etiology of heart failure.
