# Supplementary figures and images for: Myocardial Work Assessment for the Prediction of Prognosis in Advanced Heart Failure
Source: Front Cardiovasc Med. 2021 Jun 18;8:691611. doi: 10.3389/fcvm.2021.691611 (PMC8249920; doi:10.3389/fcvm.2021.691611)

**A**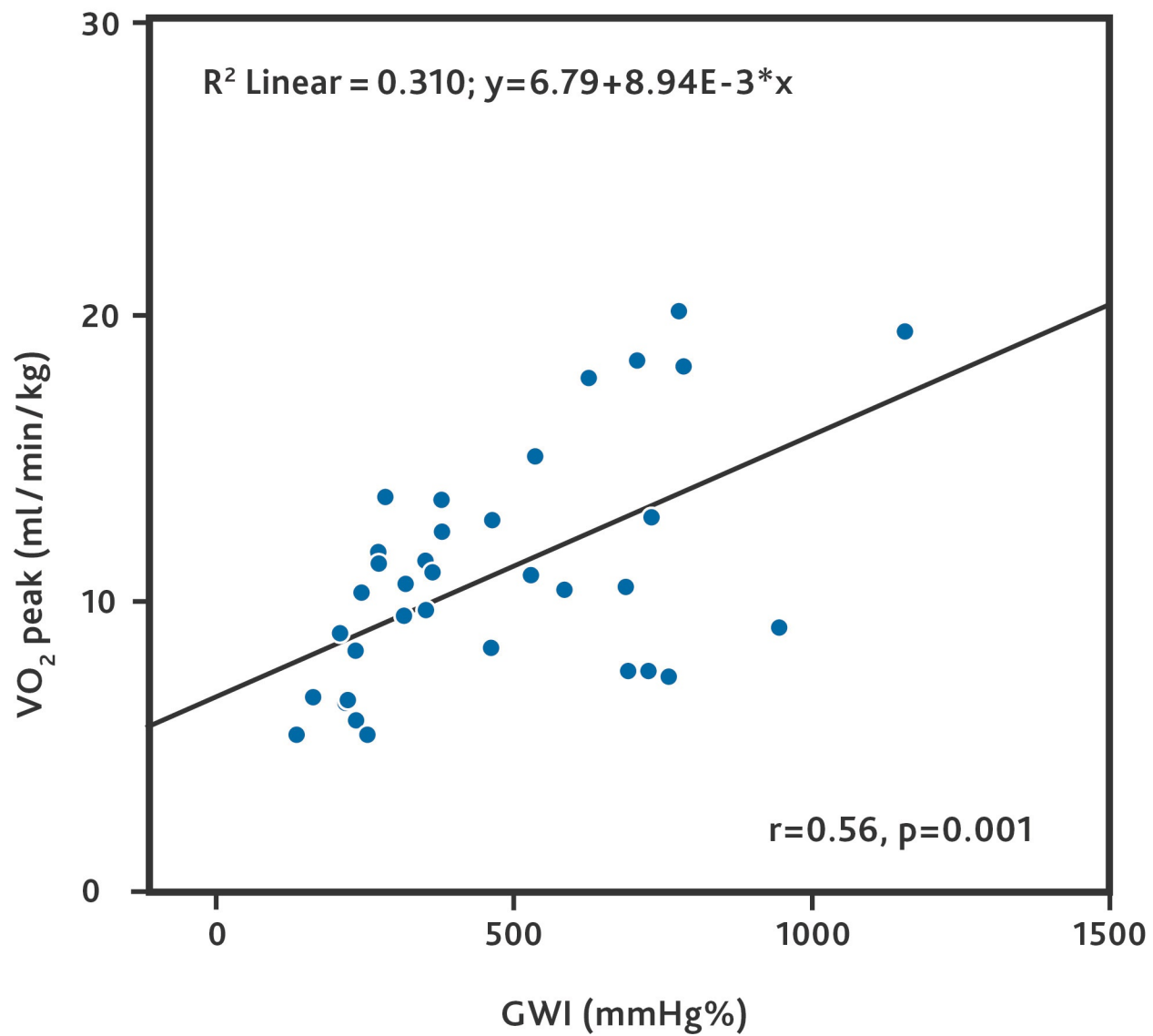**B**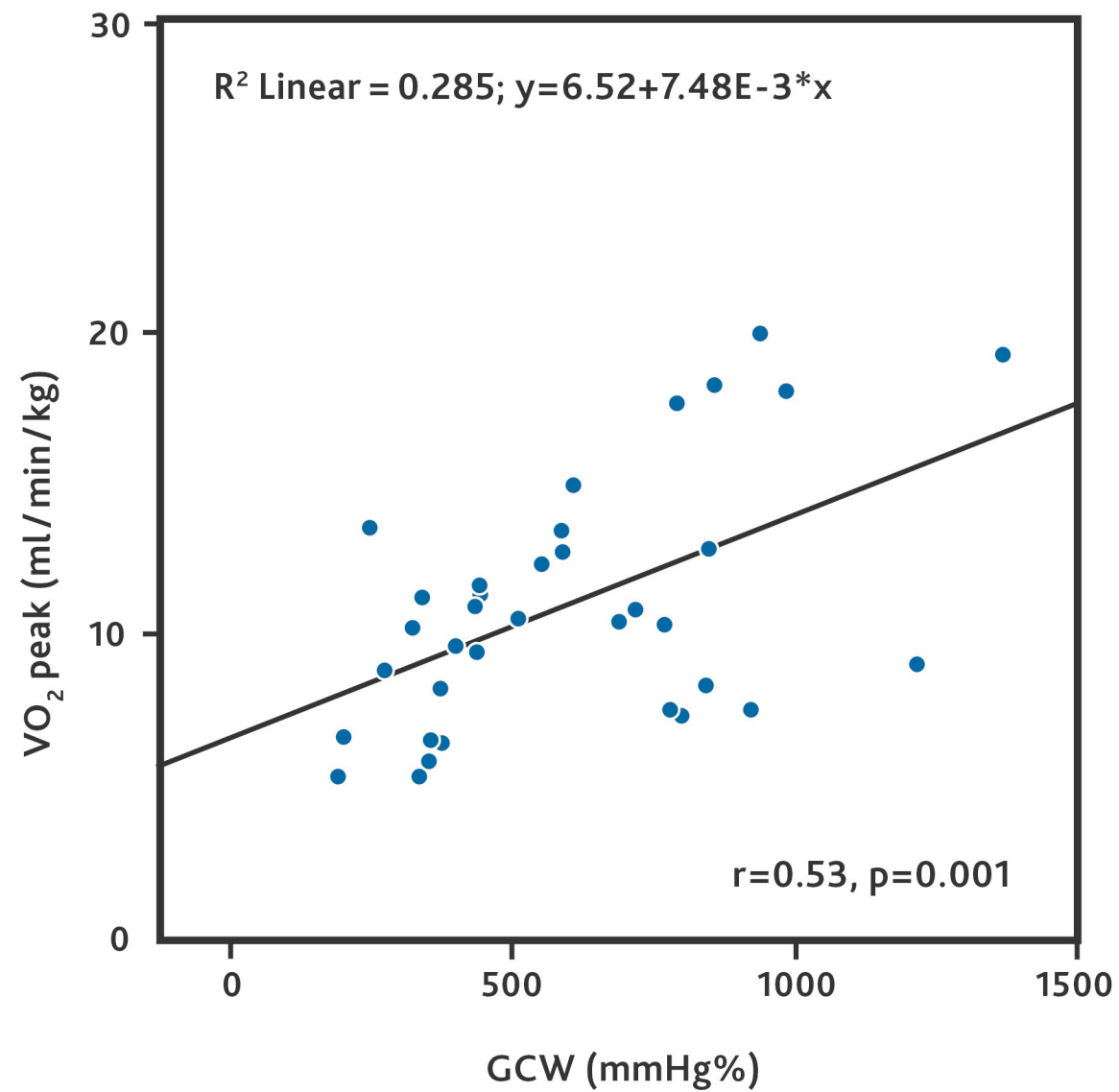

Supplement: Supplementary Figure 1 — Correlation between peak oxygen uptake and global work parameters. (A) Correlation between peak oxygen uptake (VO2 peak) and global work index (GWI). (B) Correlation between VO2 peak and global constructive work (GCW). Patients with heart failure caused by ischemic heart disease. [file Image_1.pdf]
