# Supplementary material for: Myocardial Work Assessment for the Prediction of Prognosis in Advanced Heart Failure
Source: Front Cardiovasc Med. 2021 Jun 18;8:691611. doi: 10.3389/fcvm.2021.691611 (PMC8249920; doi:10.3389/fcvm.2021.691611)
Supplement: Supplementary Table 1 — Clinical and echocardiographic parameters of patients with cardiac resynchronization therapy (CRT) device and without CRT device. [file Data_Sheet_1.docx]

**Supplemental Materials**

**Supplemental Table 1**. Clinical and echocardiographic parameters of patients with cardiac resynchronization therapy (CRT) device and without CRT device

|  | Patients with CRT  (n=40) | Patients without CRT  (n=65) | P-value |
| --- | --- | --- | --- |
| Age (years) | 57.3 ± 7.8 | 49.2 ± 12 | 0.0002 |
| Heartrate (/min) | 67 ± 8 | 71 ± 13 | 0.042 |
| Blood pressure (mmHg) |  |  |  |
| systolic | 106 ± 19 | 107 ± 17 | 0.7 |
| diastolic | 64 ± 12 | 67 ± 13 | 0.17 |
| LVEDDI (mm/m^2^) | 31.1 ± 3 | 31.2 ± 6 | 0.93 |
| LVEDVI - Simpson (ml/m^2^) | 119.5 ± 37 | 103 ± 39 | 0.035 |
| LVEF - Simpson (%) | 27.1 ± 7 | 28.3 ± 9 | 0.45 |
| SV- LVOT (ml) | 55.3 ± 14 | 51.8 ± 15 | 0.25 |
| TAPSE (mm) | 20.7 ± 4.7 | 19.5 ± 4.3 | 0.2 |
| Global longitudinal strain (%) | -6.4 ± 2.5 | -7.5 ± 3.5 | 0.07 |
| GWE (mmHg%) | 73.7 ± 10.3 | 77.8 ± 10 | 0.05 |
| GWI (mmHg%) | 524 ± 303 | 651 ± 337 | 0.055 |
| GCW (mmHg%) | 690 ± 362 | 774 ± 361 | 0.25 |
| GWW (mmHg%) | 189 ± 113 | 149 ± 73 | 0.056 |
| GPW (mmHg%) | 703 ± 356 | 788 ± 356 | 0.24 |
| GSCW (mmHg%) | 644 ± 340 | 727± 338 | 0.014 |

Values are given as mean ± standard deviation. CRT= cardiac resynchronization therapy; LVEDDI = left ventricular end-diastolic diameter index; LVEDVI = left ventricular end-diastolic volume index; LVEF = left ventricular ejection fraction; SV – LVOT = stroke volume calculated by continuous equation; TAPSE = tricuspid annular plane systolic excursion

GWE = global work efficiency; GWI = global work index, GCW = global constructive work, GWW = global wasted work; GPW = global positive work; GSCW = global systolic constructive work

**Supplemental Table 2**. Demographic, clinical, echocardiographic and outcome data according to etiology of heart failure

|  | ICM  (n=42) | DCM  (n=63) | p-value |
| --- | --- | --- | --- |
| Age (years)  (median [IQR]) | 56.5  [51-61] | 53  [41-58] | 0.02 |
| Gender |  |  | 1.0 |
| Female | 8 (19) | 13 (20.6) |  |
| Male | 34 (81) | 50 (79.4) |  |
| Body mass index (kg/m^2^) | 28.3 ± 4.6 | 28.5 ± 4.6 | 0.82 |
| Blood pressure (mmHg) |  |  |  |
| Systolic | 105 ± 18 | 107 ± 18 | 0.56 |
| Diastolic | 66 ± 12 | 66 ± 13 | 0.86 |
| NYHA class |  |  | 0.92 |
| NYHA II | 17 (40.5) | 28 (44.4) |  |
| NYHA III | 23 (54.8) | 32 (50.8) |  |
| NYHA IV | 2 (4.8) | 3 (4.8) |  |
| NT-proBNP, pg/dL  (median [IQR]) | 1193  [417-5293] | 1227  [439-2835] | 0.42 |
| Devices |  |  |  |
| ICD | 18 (42.9) | 25 (39.7) | 0.84 |
| CRT±D | 16 (38.1) | 24 (38.1) | 1.0 |
| Bundle branch block |  |  |  |
| LBBB | 5 (11.9) | 9 (14.3) | 0.78 |
| Medication |  |  |  |
| Beta-blocker | 37 (88.1) | 61 (96.8) | 0.11 |
| ACE-I | 10 (23.8) | 10 (15.9) | 0.32 |
| ARB | 5 (11.9) | 8 (12.7) | 1.0 |
| ARNI | 26 (61.9) | 45 (71.4) | 0.4 |
| Aldosterone antagonist | 35 (83.3) | 55 (87.3) | 0.58 |
| Loop diuretic | 34 (81) | 56 (88.9) | 0.27 |
| Cardiopulmonary exercise test |  |  |  |
| VO_2_ peak, ml/min/kg | 11.0 ± 4.0 | 12.5 ± 5.4 | 0.165 |
| VE/VCO_2_ slope, l/l | 37  [29-44] | 33  [28-40] | 0.09 |
| Echocardiography |  |  |  |
| LVEDDI (mm/m^2^) | 31.1 ± 5.4 | 31.2 ± 4.9 | 0.93 |
| LVEDVI – Simpson (ml/m^2^) | 111 ± 35 | 108 ± 42 | 0.68 |
| LVEF – Simpson (%) | 26.7 ± 8.3 | 28.6 ± 8.1 | 0.24 |
| SV - LVOT (ml) | 51.6 ± 14.7 | 54.1 ± 14.8 | 0.4 |
| Mitral regurgitation 2+ | 4 (9.5) | 10 (15.9) | 0.4 |
| PA pressure (mmHg) | 35.7 ± 14 | 27.4 ± 9 | 0.024 |
| TAPSE (mm) | 19.7 ± 4.2 | 20.1 ± 4.7 | 0.69 |
| E/e´ average | 18.9 ± 9 | 16.6 ± 8.5 | 0.2 |
| Global longitudinal strain (%) | -6.0 ± 2.9 | -7.8 ± 3.2 | 0.003 |
| GWE (mmHg%) | 71.3 ± 8.9 | 79.5 ± 9.8 | <0.0001 |
| GWI (mmHg%) | 496 ± 276 | 674 ± 344 | 0.006 |
| GCW (mmHg%) | 613 ± 294 | 828 ± 381 | 0.003 |
| GWW (mmHg%) | 172 ± 99 | 159 ± 88 | 0.48 |
| GPW (mmHg%) | 642 ± 306 | 831 ± 370 | 0.007 |
| GSCW (mmHg%) | 580 ± 281 | 772 ± 355 | 0.004 |
| Outcome data |  |  |  |
| Alive without event | 29 (69) | 45 (71.4) | 0.83 |
| Deceased | 4 (9.5) | 0 | 0.023 |
| LVAD | 9 (21.4) | 10 (15.9) | 0.61 |
| HTX | 0 | 8 (12.7) | 0.02 |

Values are given as a number (percent) or mean ± standard deviation except where otherwise indicated.

ICM = heart failure caused by ischemic heart disease; DCM = dilated cardiomyopathy; IQR = interqartile range; NYHA = New York Heart Association; BNP = brain natriuretic peptide; ICD = implantable cardioverter-defibrillator; CRT±D = cardiac resynchronization therapy with or without defibrillator; LBBB = left bundle branch block; ACE-I = angiotensin-converting enzyme inhibitor; ARB = angiotensin II receptor blocker; ARNI = angiotensin-receptor neprilysin inhibitor; peak VO2 = maximum oxygen uptake; VE/VCO2 = ventilation-carbon dioxide output relation

LVEDDI = left ventricular end-diastolic diameter index; LVEDV = left ventricular end-diastolic volume; LVEDVI = left ventricular end-diastolic volume index; LVEF = left ventricular ejection fraction; SV – LVOT = stroke volume calculated by continuous equation; PA = pulmonary artery calculated from tricuspid reg. peak velocity; TAPSE = tricuspid annular plane systolic excursion

GWE = global work efficiency; GWI = global work index, GCW = global constructive work, GWW = global wasted work; GPW = global positive work; GSCW = global systolic constructive work

LVAD = left ventricular assist device, HTX = heart transplantation
